# Development and characterisation of an expressed sequence tags (EST)-derived single nucleotide polymorphisms (SNPs) resource in rainbow trout

**DOI:** 10.1186/1471-2164-13-238

**Published:** 2012-06-13

**Authors:** Mekki Boussaha, René Guyomard, Cédric Cabau, Diane Esquerré, Edwige Quillet

**Affiliations:** 1 INRA, UMR 1313 Génétique Animale et Biologie Intégrative, 78350, Jouy-en-Josas, France; 2 INRA, SIGENAE UR83 Recherches Avicoles, 37380, Nouzilly, France; 3 INRA, UMR 444 Laboratoire de Génétique Cellulaire Plateforme GET, Castanet, Tolosan, France

## Abstract

**Background:**

There is considerable interest in developing high-throughput genotyping with single nucleotide polymorphisms (SNPs) for the identification of genes affecting important ecological or economical traits. SNPs are evenly distributed throughout the genome and are likely to be functionally relevant. In rainbow trout, *in silico* screening of EST databases represents an attractive approach for *de novo* SNP identification. Nevertheless, EST sequencing errors and assembly of EST paralogous sequences can lead to the identification of false positive SNPs which renders the reliability of EST-derived SNPs relatively low. Further validation of EST-derived SNPs is therefore required. The objective of this work was to assess the quality of and to validate a large number of rainbow trout EST-derived SNPs.

**Results:**

A panel of 1,152 EST-derived SNPs was selected from the INRA Sigenae SNP database and was genotyped in standard and double haploid individuals from several populations using the Illumina GoldenGate BeadXpress assay. High-quality genotyping data were obtained for 958 SNPs representing a genotyping success rate of 83.2 %, out of which, 350 SNPs (36.5 %) were polymorphic in at least one population and were designated as true SNPs. They also proved to be a potential tool to investigate genetic diversity of the species, as the set of SNP successfully sorted individuals into three main groups using STRUCTURE software. Functional annotations revealed 28 non-synonymous SNPs, out of which four substitutions were predicted to affect protein functions. A subset of 223 true SNPs were polymorphic in the two INRA mapping reference families and were integrated into the INRA microsatellite-based linkage map.

**Conclusions:**

Our results represent the first study of EST-derived SNPs validation in rainbow trout, a species whose genome sequences is not yet available. We designed several specific filters in order to improve the genotyping yield. Nevertheless, our selection criteria should be further improved in order to reduce the observed high rate of false positive SNPs which results from the occurrence of whole genome duplications.

## Background

International genome initiatives have resulted in draft sequences of the genome of several farm animals (cattle, pig, chicken, and horse) and of model fish species (zebrafish (*Danio rerio*), medaka (*Oryzias latipes*), stickleback (*Gasterosteus aculeatus*), takifugu (*Takifugu rubripes*), and tetraodon (*Tetraodon nigroviridis*)). Whole genome sequencing are currently underway for a number of aquaculture species: rainbow trout (*Oncorhynchus mykiss*), Atlantic salmon (*Salmo salar*), Nile tilapia (*Oreochromis niloticus*), Asian seabass (*Lates calcarifer*), European seabass (*Dicentrarchus labrax*), channel catfish (*Ictalurus punctatus*) and common carp (*Cyprinus carpio*). At the same time, high-throughput genomic tools have been developed, improving the description of genomic structure and function.

Projects associated with genome sequencing activities using different breeds from the same species have provided the opportunity to discover hundreds of thousands of potential single-base changes, also known as single nucleotide polymorphisms (SNPs) or short insertion/deletion mutations (indels). The bi-allelic nature of SNPs makes them less informative than microsatellites. Nevertheless, SNPs are considered as a highly reliable and valuable molecular marker system for genotyping and selective breeding because of their omnipresence throughout the entire genome, both within gene coding and non-coding regions.

SNPs in gene coding sequences can be either synonymous (silent polymorphism) or non-synonymous (replacement polymorphism). They are of particular interest to study the genetics of expressed genes and to map functional traits. Synonymous SNPs may alter RNA secondary structures and can affect protein conformation and function [1]. Non-synonymous SNPs can potentially have deleterious functional effects because they lead to changes in amino acid sequences and possibly affect protein structure and function [2,3].

SNPs in non-coding regions can occur in introns, promoters, intergenic sequences, and in 5'- or 3'-untranslated regions. They may alter gene expression by affecting gene splicing, transcription factor binding, mRNA degradation, or non-coding RNA sequences.

Over the last decades, large-scale SNP production initiatives have been associated with the development of high-throughput genotyping technologies that facilitate the simultaneous analysis of hundreds of thousands of SNPs. These low-cost but highly reliable assays have permitted fine-scale gene mapping and candidate gene association studies for complex traits in several species such as humans [4], mouse [5] chicken [6], cattle [7] and sheep [8].

In species whose complete genome sequences are not yet accessible, the increasing availability of expressed sequence tags (ESTs) represents an alternative *in silico* strategy for *de novo* SNP identification. This approach does not require any additional bench work, offers a low cost source of SNPs, and has been recently used in a few aquaculture species such as blue and channel catfish species [9], and salmonids [10-13]. Moreover, EST-derived SNPs are considered as gene-derived SNPs since they are located within gene coding and 3′-UTR regions and they can lead to the identification of quantitative trait nucleotides (QTN) [14].

However, the usefulness of EST-derived SNPs remains putative until their true informativity (sequence polymorphism) and duplication status have been checked with genomic DNA in the populations of interest. Although it is possible to use base quality values to discern true allelic variations from sequencing errors, validation is a key step for detection of true SNPs [15]. This is generally carried out by genotyping several population samples with a subset of the EST-derived SNPs [10].

Rainbow trout is the most widely cultivated cold freshwater fish in the world. It has great potential for aquaculture and recreational sport fisheries. In addition to its commercial interest, rainbow trout is also a model species for a wide range of genome-related research activities [16].

The rainbow trout haploid genome size was estimated to be between 2.4 and 3.0 × 109 bp [17,18]. A common ancestor of rainbow trout and other salmonids has undergone a fourth whole-genome duplication (4R WGD) event about 25 to 100 million years ago, which was followed by a period of re-diploidization resulting in a semi-tetraploid state [19]. It has been estimated that up to half of the loci are still duplicated [20]. Although the tetraploidization event increases the genome complexity, it also makes the salmonids an attractive model to study the mechanisms behind the whole-genome duplication event and the subsequent reduction of one of the two copies of the duplicated gene(s).

Both the interest brought into rainbow trout as a research model and the need for its genetic improvement for aquaculture production efficiency and product quality led to the development of several genomic resources for this species. Meanwhile, great efforts have been and are still devoted to the development of SNP genetic markers [21-23].

Previous efforts using reduced representation libraries [22] and reference transcriptome datasets [24] resulted in the production of up to 47,000 and 58,000 putative SNPs, respectively. A subset of 384 randomly selected SNPs were genotyped on individual fish and 184 (48 %) were validated [22]. The observed low validation rate could be partly explained by the presence of paralogous sequences with allelic variation which resulted in the production of false positive SNPs.

Finally, these putative SNPs were not yet publicly available. Therefore, EST-derived SNPs could represent an alternative and complementary in silico approach to assess the quality and to validate larger numbers of SNPs. These resources will add to the already available 184 SNPs validated from the reduced representation libraries study.

Miller and co-authors [23] have also used the RAD (Restriction site Associated DNA) sequencing technology for low density SNP genotyping and reported the construction of a high-resolution linkage map containing 4,563 markers. However, the flanking sequences for these SNPs were only 68 nucleotides long and thus may not be suitable for the design of high-throughput genotyping assays, such as the Illumina assays. Retrieving longer flanking sequences suitable for high-throughput genotyping studies using these RAD-associated markers will need additional information on the whole genome sequence. Efforts are in progress in France and USA [25,26] to provide a rainbow trout reference genome sequence in the near future. Nevertheless, in both cases, aiming at facilitating the assembly step, the sequencing was performed using a doubled haploid homozygous DNA sample which hinders the identification of new SNPs.

Mining EST datasets remains an attractive alternative approach for *in silico* SNP identification in rainbow trout. Up to 31,121 *in silico* EST-derived SNPs are currently available at the INRA Sigenae database ( http://www.sigenae.org/). However, they do not provide any information neither on their true informativity nor on their duplication status. Therefore, it is necessary to validate the status of these markers. Validation of rainbow trout EST-derived SNPs in a large number of populations will not only allow to identify fully informative true SNPs but also will highlight the proportion of informative SNPs shared across different populations, a crucial information to efficiently design future rainbow trout specific SNP chips. These new tools will contribute to studies on population genetics and will facilitate quantitative trait loci (QTL) identification, and marker assisted selection.

In the present study, a panel of 1,152 EST-derived SNPs was selected from the Sigenae SNP database and were subsequently assayed for allelic variation in several rainbow trout population samples using the Illumina GoldenGate assays. Successfully validated EST-derived SNPs were used to analyse the genetic diversity in three bisexually reproducing experimental stocks and a collection of doubled haploid (DH) clones and to update the INRA linkage map by integrating 223 new markers.

## Methods

### Selection of SNPs for validation

The INRA Sigenae rainbow trout EST-derived SNP database ( http://www.sigenae.org/; restricted access) was used to select a validation SNP panel. A public version of this release will be available in the near future. Almost 31,121 SNPs were produced by assembling EST sequences. Briefly, several stringent filters were used to improve the quality of predicted SNPs: (1) the value of the local depth at the polymorphic position must be at least equal to 7; (2) the 4 bases flanking regions around the SNP position need to be exactly conserved within the aligned sequences; (3) the minimal number of sequences with the lowest represented base must be at least equal to 3; (4) gaps on consensus sequences were ignored; and (5) N or gaps on sequences were ignored.

**Figure 1:**
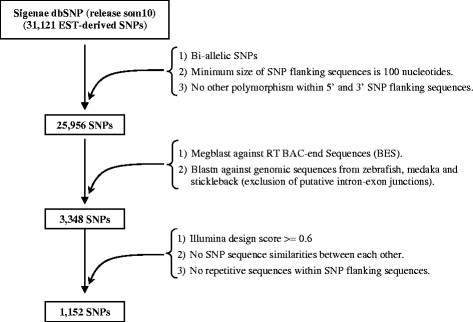
**Selection of the validation panel.** Filters used to select EST-derived SNPs for validation from the INRA Sigenae SNP database release som10 were summarized.

Several selection filters (Figure 1) were applied in order to select a panel of 1,152 EST-derived SNPs for validation: (1) in order to meet the requirements for probe design constraints for the Illumina genotyping platform, all SNPs with less than 60 nucleotides between two neighbouring SNPs and with flanking sequences less than 100 nucleotides long were removed; (2) in order to overcome problems due to exon-intron junctions, the SNP flanking sequences were aligned against rainbow trout BAC-end sequences [27] using megablast tools and against zebrafish, medaka, and stickleback genomic sequences using blastn tools. All SNP sequences with an alignment length equal to the flanking sequence length were selected for further analysis. The filtered SNP sequences were then submitted to Illumina to assess their design quality. Only those showing a minimum quality score of 0.6 were further filtered against sequence similarities between each other and against the presence of repetitive sequences. After applying the above filters, a panel of 1,152 EST-derived SNPs was constructed and was used to genotype a large number of rainbow trout individuals.

### DNA sources

Two hundred and fifty seven DNA individual rainbow trout were genotyped for each of the 1,152 SNPs using the Illumina GoldenGate assay. These include 37 INRA doubled haploid (DH) individuals, DNA from 10 DH individuals from various origins provided by Dr Gary Thoorgard (ARS), 20 individuals from the INRA synthetic reference strain (INRA-SY), 20 individuals from the INRA spring spawning strain (INRA-SP), and DNA from 44 individuals from five NCCCWA mapping families [28] provided by Dr Yniv Palti (ARS). The two INRA reference mapping families (two parents with four grandparents and 120 DH progeny) were also included. DNA was isolated from fin clips stored in 95 % ethanol, according to the protocols previously described [29].

INRA rainbow trout fin clips were collected from euthanized and/or anesthetized fish elevated at the INRA fish farm facilities. Under French regulation, the INRA facilities are authorized for experimental activities and both the staff of the facilities and scientists have personal authorization to conduct animal experimentations. All animal manipulations were done according to the good animal practice as defined by the French Direction of Veterinary Services.

### Genotyping

High-throughput genotyping reactions were performed at the INRA genomics GET PlaGe core facility, using the Illumina GoldenGate BeadXpress systems, according to the manufacturer's protocol [30]. SNPs with an Illumina design score above 0.6 were retained for further analysis. Oligonucleotides were designed, synthesised, and assembled into three custom oligo pooled assays (OPA) by Illumina Inc.

Genotype clustering was performed using the GenomeStudio software (Illumina Inc.). GenCall and GenTrain quality scores for each genotype were generated. A GenCall score cutoff of 0.25 was used to determine valid genotypes at each SNP and the retained SNPs had to have a minimum GenTrain score of 0.25 (a stringent criterion that is used in human genetic studies) [31]. Clusters were visually inspected to ensure high quality data. Genotype calls were exported as spreadsheets from the GenomeStudio software for further analysis.

### Population structure

The STRUCTURE software [32] was used to assess the population structure. This program implements a model-based clustering method to infer population structure using genotype data of unlinked markers. We used the admixture model and correlated allele frequency version of the STRUCTURE program [33]. To choose the most likely number of clusters modelling the data, several analyses were performed, for a number of fixed subgroups K (number of populations) from 1 to 5. Each analysis involved five independent runs with a burn-in period of 50,000 and 200,000 iterations for the likelihood estimation. The best K value which corresponds to the K with the highest Delta K score was determined using a non parametric test as previously described [34]. This test uses an ad hoc quantity (delta K) calculated based on the second order rate of change of the likelihood (delta K).

### Functional annotations of polymorphic SNPs

Both contig and SNP allele sequences were analysed for gene content by blastx using the ENSEMBL non redundant protein databases for zebrafish (Danio_rerio.Zv9.64.pep.all.fa).

Blastx searches were carried out using an e-value cut off of 1e-5. The blastx search results were filtered to remove non specific homologies using the following filtration steps: (1) the Ensembl protein ID in the blastx results were renamed by their corresponding Ensembl gene ID (since each gene may encode several peptides due to alternative splicing), (2) for each sequence read (query ID) with a gene hit (subject ID), results were filtered to keep only the hits with the minimal e-value score; and (3) sequence reads with several hits having the same minimal e-value were further filtered to keep the hits with the highest HSP (high-scoring segment pairs; calculated as the product of % identity multiplied by alignment length). Only SNP sequences and their corresponding contig sequences having a gene hit were used for further analysis.

For each contig read, query start and query end positions were used to retrieve corresponding contig sequences between these two values. DNA sequences were then translated and the translation product was used to construct a specific RT peptide database. Both SNP allele sequences were then analyzed for synonymous/non-synonymous SNPs by blastx using the produced RT peptide database. For synonymous SNPs, both allele sequences should result in a perfect match with a given peptide sequence (100 % identity). For non-synonymous SNPs, one allele sequence should result in a perfect match and the other should present only one amino acid mismatch.

Finally, we assessed the deleterious effect of non-synonymous SNPs using SIFT (Sorting Intolerant From Tolerant) program (sift.jcvi.org/). Prediction was carried out using the SIFT sequence tool through PSI-Blast searches against UniProt - SwissProt databases (release 57.15, April 2011). Median conservation of sequences was fixed to 3.0 and hits showing more than 90 % identity to the query sequence were removed.

### Linkage map construction

Linkage groups were constructed with CARTHAGENE [35] and optimized with the annealing option (argument values: 15, 300, 0.1, 0.5) (see Carthagene help for argument meaning). Since interference is close to 1 in salmonids, we used the percentage of recombination as mapping function. Graphical representations were obtained with MAPCHART [36].

## Results and Discussions

### Sigenae SNP database characterization and selection of a subset for validation

The Sigenae rainbow trout EST-derived SNP database ( http://www.sigenae.org) contains 31,121 putative SNPs identified in 13,374 EST contigs (Table 1). The total length of contig sequences was estimated to be 2.23 Mb with an average contig length of 1,889 bp ranging from 134 to 9,913 bp. This corresponds to one SNP every 716 bp which is slightly higher than previously reported frequencies from a panel of SNPs obtained using the RAD sequencing approach [23]. The average sequence coverage was estimated to be 12.7 sequence/contig and ranged from 7 to 466 sequences/contig.

**Table 1:** Distribution of SNPs in EST contigs

**Number of SNPs / contigs**	**Sigenae db SNP Number of contigs**	**Validation panel Number of contigs**
1	6570	151
2	2962	141
3	1549	122
4	850	88
5	502	67
6	298	58
7	199	46
8	119	33
9	96	33
10	56	24
> 10	173	100
**Total**	**13 374**	**863**

Total number of EST contigs containing one or more SNPs were indicated for both the initial database and the validation panel.

Almost 83 % of the EST-derived SNPs were identified from contigs containing one to five SNPs (Table 1). The mean minor sequence frequency among all SNPs was 0.37 ± 0.1 (SD), while the mean observed heterozygosity based on sequence coverage at the polymorphic site was 0.45 ± 0.07, and the mean PIC (polymorphism information content) was 0.34 ± 0.04 (Additional file 1: Sheet 1).

After application of several selective filters (Figure 1) designed to improve the expected yield of genotyping, a subset of 1,152 SNPs was selected for our study (Additional file 2). Three OPA (Oligo Pool Assays) each comprising 384 SNPs were designed and were called the validation panel. SNPs from the validation panel were identified in 863 contigs, of which 66 % contain one to five SNPs (Table 1). The mean minor sequence frequency among the validation panel was estimated to 0.36 ± 0.1 (SD), while the mean observed heterozygosity based on sequence coverage at the polymorphic site was 0.44 ± 0.08 and the mean PIC was 0.34 ± 0.05 (Additional file 1: Sheet 2) which were very close to those calculated from the Sigenae SNPs database.

### SNP validation

The efficiency of the selection approach and the relevance of the resulting SNPs were assessed by genotyping the validation panel in a number of rainbow trout doubled haploid (DH) and standard individuals from three different domestic populations.

Assays were developed for 1,152 putative EST-derived SNPs, out of which 958 (83 %) were successfully genotyped (Table 2 and Additional file 3: Sheets 1–5) while genotyping failed for 194 SNPs (17 %). These did not either cluster well according to genotype or failed to amplify most probably because of the sequence complexity or the presence of polymorphisms within flanking sequences or failed manufacture with Illumina. These were considered "failed assays". Out of the 958 successfully genotyped SNPs, 55 % were selected from contigs containing no more than 5 SNPs and the overall proportion of successfully genotyped SNPs over those from the validated panel did not depend on the SNP content in EST contigs (Table 3).

**Table 2:** Minor allele frequency of validated SNPs

**SNPs**	**DH lines**	**INRA SP**	INRA SY*	**NCCCWA**	3 pop.*
**Monomorphic**	352	390	367	351	346
**Potentially duplicated**	262	272	268	266	262
**True**	344	296	322	341	350
*MAF < 0.05*	27	7	11	22	21
*0.05 > = MAF < 0.10*	30	33	30	36	26
*0.10 > = MAF < 0.20*	58	55	51	73	74
*0.20 > = MAF < 0.30*	63	55	71	56	66
*0.0 > = MAF < 0.40*	85	68	77	67	66
*0.40 > = MAF < 0.50*	81	78	82	87	97
**Total count**	**958**	**958**	**957**	**958**	**958**

SNPs were clustered into different categories based on their observed MAF in rainbow trout DH individuals and in the three population samples analysed

* one snp was not considered for MAF calculation because of genotyping failure in all samples.

**Table 3:** Distribution of the genotyped SNPs in contigs

**Number of SNPs/contig**	**Number of SNPs in:**				
	**Validation Panel**	**Successfully genotyped**	**Monomorphic**	**Heterozygous**	**True**
1	151	133	34	13	86
2	154	126	39	21	66
3	141	115	34	24	57
4	98	79	30	20	29
5	91	69	25	20	24
6	81	72	24	30	18
7	70	53	29	14	10
8	48	39	18	11	10
9	45	40	19	12	9
10	38	29	7	12	10
> 10	235	203	87	85	31
Total	1152	958	346	262	350

The number of SNPs identified within each contig type were summarized for the validation panel and for successfully genotyped, monomorphic, heterozygous and for true SNPs.

Almost 36 % of the successfully genotyped SNPs were homozygous in all samples (i.e. only one SNP variant was identified in all individuals). These were incorrectly identified as SNPs since EST sequencing presents a high rate of sequencing errors resulting in the identification of pseudo-SNPs. Some of these SNPs may also correspond to rare polymorphisms that were not present in the population samples genotyped in this study. ESTs are issued from a wide variety of tissues usually collected from a limited number of individuals and genetically constrained populations and may represent a bias in true allelic variations. The effectiveness of EST resources to detect *in silico* SNPs highly depends on the collection of tissues used and the diversity of the target samples as well as on how well this diversity is represented within the EST databases used for SNP identification [37,38].

Two hundred and sixty two SNPs (27 %) revealed paralogous sequences as all samples, including DH individuals, were heterozygous. Out of these, 63 % were identified in contigs containing at least six SNPs/contig (Table 3). Since up to 50 % of the rainbow trout genome could have retained duplicated regions, the high proportion is most probably due to the assembly of duplicated gene sequences which could result in the production of paralogous site variants (PSVs). PSVs are sequence differences between two paralogous loci but the substitution does not segregate within either locus and were considered false positive SNPs. Similar observations were obtained with Atlantic salmon [10,12,13].

**Figure 2:**
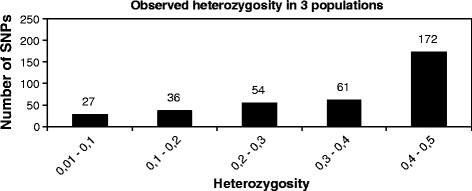
**Distribution of observed heterozygosity for true SNPs in three populations.** SNPs were clustered into categories based on their observed heterozygosity values.

**Figure 3:**
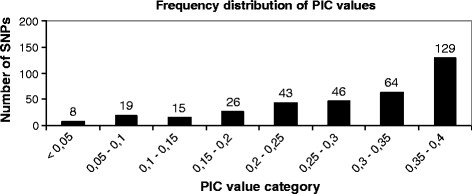
**Frequency distribution of PIC values across the three genotyped populations.** SNPs were clustered into categories based on their observed PIC values.

Finally, 37 % (350) of the successfully genotyped SNPs were polymorphic and reliably scored, and thus were considered as true SNPs (Table 2). They were identified in 321 contigs. The yield of true SNPs decreased with the number of SNPs/contig and almost 75 % of those true SNPs were identified from contigs containing no more than five SNPs/contig (Table 3). The mean of observed minor allele frequency (MAF) among true SNPs was 0.27 ± 0.14 (SD), while the mean observed heterozygosity (Figure 2**)** across loci was 0.35 ± 0.14, and the mean PIC (Figure 3**)** was 0.28 ± 0.1 (see also Additional file 3: Sheet 6). Since observed heterozygosity and PIC rates are near the maximum theoretical values for a bi-allelic marker, we can conclude that the validation panel is highly informative for this type of markers. These SNPs are of particular interest for linkage analysis since we can easily follow up their segregation from one generation to another.

### Population assignment

Three domestic populations of different origins (INRA SP and SY strains and NCCCWA population) were used in this study. This offers the opportunity to determine whether a Bayesian clustering software such as STRUCTURE could detect the underlying genetic populations among all analysed samples using the observed SNP genotypes only.

**Figure 4:**
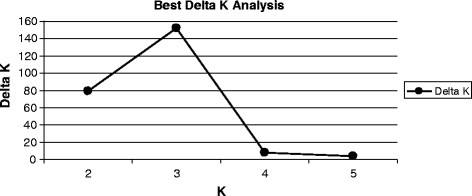
Prediction of the best value of K. Delta K analysis was performed as previously described (Evanno, 2005) in order to predict the best value of K.

**Figure 5:**
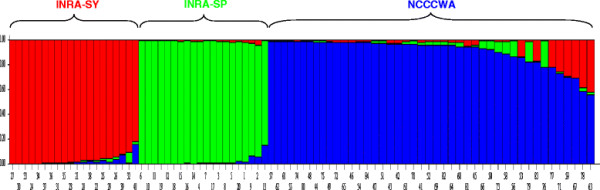
**Genetic population structure predicted by STRUCTURE software.** Genetic population structure was inferred with the structure software using K = 3, a burn-in period of 50,000 200,000 iterations for the likelihood estimation. Individuals 1 to 20 correspond to the INRA-SP population, individuals 21 to 40 correspond to the INRA-SY population and individuals 41 to 84 correspond to the NCCCWA population.

We first used the non parametric approach [34] to infer the optimal number of populations (true K value). Inference of the best K using the delta K method revealed a clear peak at K = 3 (Figure 4 and Additional file 4) which corresponds to the true number of populations used in the present study. For this K value, STRUCTURE software successfully sorted individuals into three main groups which corresponded entirely to the discrete three main populations sampled in the study (Figure 5). These results should assist the design of SNP- rather than microsatellite-based studies to detect population structure in a larger collection of rainbow trout.

Even though microsatellites have higher allele diversity, the frequent occurrence of mutations following a stepwise mutation model within these markers may lead to homoplastic alleles which may represent a significant problem in population genetics [39]. SNPs are considered biallelic and individual SNP loci have lower information content than microsatellite. However, they are highly frequent within genomes, have low mutation rates and these features allow reconstituting highly informative and non homoplasic haplotypes. With the advent of high throughput genotyping strategies, SNPs open new avenues in population genetics such as association studies in natural populations. From a practical point of view, they offer more rapid, highly automated and more reliable genotyping which are also useful properties for population inference analysis.

### Functional annotation of true SNPs

To assign putative functions to the 350 true SNPs, we performed blastx searches of both SNPs and corresponding EST contig sequences against the ENSEMBL zebrafish non redundant peptide database.

Blastx search results made it possible to assign putative functions to 321 contig sequences (Additional file 5: Sheet 1). Of these, 279 contig sequences showed unique gene hits, 35 contig sequences showed unique gene hits but multiple alignment positions and seven contig sequences had multiple paralogous gene hits.

Blastx searches using true SNP sequences revealed that 339 markers resulted in the same gene hits as those found with their corresponding contig sequences (Additional file 5: Sheet 2). Among these, 11 SNPs did not show any homology search results and 12 SNPs matched with target regions in gene hits different from those found with the corresponding contig sequences. These 23 markers were excluded from the SNP panel used for synonymous/non-synonymous prediction analyses. Out of the remaining 327 SNPs, 28 were identified as non-synonymous and four of these substitutions were predicted to affect protein functions (Additional file 5: Sheet 3). This is particularly important since they could be considered as valuable sources of candidate gene polymorphisms underlying important traits leading to the identification of causative genes. However, these predictions were conducted using computational tools and functional data analyses are therefore needed to validate the most likely effect of these substitutions on the protein functions.

Non-synonymous SNPs are of particular interest because they are more likely to alter the biological function of a protein. They are suitable markers for comparative genome mapping and for marker-assisted selection of economically important traits [40,41].

Even though synonymous SNPs have long been considered as silent substitutions, they are also of particular interest since they can alter RNA secondary structures and affect regulation of gene expression [1].

### Transitions/transversions ratio

About 74.1 % of SNPs from the validation panel were A ↔ G and C ↔ T transitions representing 28.2 % and 45.9 of the total SNPs, respectively (Table 4). For both true and synonymous SNPs, observed transition over transversion (Ts/Tv) ratios were (3.48) and (4.07), respectively. In addition, the Ts/Tv ratio was found to be higher in synonymous (4.07) than in non-synonymous SNPs (1.15).

**Table 4:** Observed occurrence of the different SNP types (transitions and transversions)

	Type	Validation panel	polymorphic SNPs	Syn. SNPs	Non Syn. SNPs
Transition (Ts)	AG	325	88	71	11
	CT	529	184	169	4
Transversion (Tv)	AC	98	28	22	5
	AT	49	14	8	3
	CG	58	16	12	3
	GT	93	20	17	2
TOTAL		1152	350	299	28
% ratio Ts/Tv		2.87	3.48	4.07	1.15

On average, an excess of transitions was observed in this study, which is believed to be attributable to the abundant hypermutable methylated dinucleotide 5′-CpG-3 [42]. One probable explanation would be the high spontaneous rate of deamination of 5'-methylated cytosines (5mC) at CpG di-nucleotides to thymidine (C↔T) SNPs and (G↔A) on the complementary strand [43].

Synonymous SNPs are more often transitions than transversions. It is generally agreed that degeneracy in the genetic code and the stronger selection pressure against non-synonymous substitutions account for the observed increase in the relative frequencies of transitions over transversions [44].

It is difficult to draw general conclusion from the smallnumber of non-synonymous substitutions recorded here. However, transition and transversion classes tended to occur at similar levels (Table 5). Both transition and transversion classes can result in amino acid substitutions, but the biochemical differences in the corresponding protein products tend to be greater for transversions [45].

**Table 5:** Distribution of SNPs within INRA rainbow trout microsatellite-based linkage map

**Linkage group**	**Total SNPs**	**Linkage group**	**Total SNPs**
GL1	8	GL16	9
GL2	10	GL17	0
GL3	9	GL18	5
GL4_25	8	GL19	6
GL5	3	GL20	11
GL6	4	GL21	11
GL7	8	GL22	5
GL8	13	GL23	11
GL9	12	GL24	7
GL10	6	GL26	3
GL11	3	GL27	17
GL12	16	GL29	9
GL13	6	GL30	2
GL14	5	GL31	9
GL15	7	**Total**	**223**

The number of SNPs that were integrated in each rainbow trout linkage group (GL) were indicated.

### Integration of EST-derived SNPs in the INRA RT linkage map

Two unrelated reference families (two parents with four grandparents and 120 progeny) were successfully genotyped with the 350 true SNPs. Out of these, 120 SNPs were not polymorphic in the two reference families and were therefore excluded from the final panel for linkage mapping. Segregation pattern analyses of the remaining 230 SNPs revealed seven SNPs showing non Mendelian inheritance. These were excluded from the final panel for linkage mapping. The remaining 223 SNPs were successfully positioned on the microsatellite-based linkage map (Additional file 6). The number of SNPs that were assigned to each linkage group (RT) varied from 0 (RT17) to 17 (RT27). Linkage groups RT04 and RT25 were unlinked, but were artificially merged to form a metacentric linkage group, as previously reported [28,46].

One hundred markers (29 %) were identified within duplicated regions or adjacent to duplicated markers delimiting known duplicated regions of rainbow trout (Additional file 7). Four SNPs were found duplicated, out of which three pairs (snp_BX855659_100/1 and /2, snp_CX719996_684/1 and /2, snp_F3GS49K01ARLCI_353/1 and /2) were successfully mapped to the expected homeologous groups (Additional file 6). The fourth pair (snp_BX078786_199/1 and /2) were assigned to linkage groups RT01 and RT19, which did not show any homology in previous studies [28,29,46]. Additionally, three pairs of SNPs (snp_BX869590_239 and snp_CA387137_459, snp_GBPNQUD01ALF2N_185 and snp_GBPNQUD01BTTCT_137, snp_F3GS49K01CUTTZ_151 and snp_F3GS49K02I3USJ_458) were originally identified within the same EST contigs. For each pair, one SNP was assigned to a linkage group and the second one to the homeologous group (RT06 and RT30, RT12 and RT16 and RT07 and RT15, respectively). These seven markers were true but duplicated SNPs and were considered multisite variants (MSVs). Since up to 50 % of the rainbow trout *pseudotetraploid* genome still retains duplicated regions, this can lead to the production of MSVs. These are SNPs within paralogous loci with one locus fixed (monomorphic) and the other one polymorphic. MSVs can be used for both linkage and association studies and they can provide information on two paralogous loci resulting from the latest WGD event.

## Conclusions

One of the main objectives of rainbow trout genomic research is the development of rapid, accurate and automated genotyping systems for sequence variations influencing economically important traits. Developing larger sets of SNP markers for genome analyses in rainbow trout will facilitate fine QTL mapping and will improve the identification and exploitation of genes affecting important traits and enable selective breeding through genomic selection.

In the lack of a rainbow trout reference genome, EST resources represent an attractive approach for *in silico* SNP identification. In this study, we have assessed the quality of 1,152 rainbow trout EST-derived SNPs by genotyping several population samples using the Illumina GoldenGate BeadXpress assays. High-quality genotype data were obtained from 958 SNPs representing a genotyping success rate of 83.2 %. Polymorphism information was validated for 350 (36.5 %) rainbow trout EST-derived SNPs.

Almost two thirds of the successfully genotyped SNPs were considered as either false SNP (homozygous in all individuals) or duplicated heterozygous in double haploid individuals, expected to be all homozygous. This higher failure rate is most probably due to EST sequencing errors or the assembly of duplicated gene sequences, which can lead to the production of MSVs (true but duplicated SNPs) and PSVs (false positives). Although MSVs and PSVs can provide information on paralogous loci resulting from the latest WGD event in rainbow trout, their presence can hinder the use of SNP arrays in genome-wide association and should be avoided in population genetics studies. MSVs are of particular interest since they can be used for both mapping, linkage, and for association studies. However, since these markers are not easily interpreted, they were either discarded or their genotypes were incorrectly assigned. Since up to half of the rainbow trout genome still retains duplicated regions, calling and assigning MSVs to the correct paralogous loci become difficult and hinder genotype calling and genetic mapping. Moreover, currently available standard genotyping software programs have not been designed to automatically deal with this type of polymorphisms. It is noteworthy that nearly all the SNPs which were mapped in the duplicated regions were assigned to the same linkage group suggesting that, in most cases, only one of the two duplicated sites could be genotyped. This is particularly important for most of populations genetics studies where the same set of markers must be analysed.

The Illumina GenomeStudio software was recently updated and provides the GT module v2010/v1.8 which allows assigning genotypes in tetraploid genomes. However, users need to perform manual clustering of genotype calls for polyploid loci. This is unrealistic to accomplish when thousands of genotyped markers are analysed simultaneously. Consequently, an accurate and automated genotyping system for calling polyploid genotypes is highly desirable. A recent genotyping software, beadarrayMSV, has been designed and was used for genotype calling and mapping of MSVs in Atlantic salmon [47]. Nevertherless, this tool was rather useful for manipulating data genotyped using Illumina Infinium BeadArrays (Illumina Inc.) rather than GoldenGate data and therefore could not be used in the present study.

Combining the identification and validation of MSV markers with regular SNP polymorphisms in rainbow trout will allow the identification of fully informative genetic markers in this species and will provide valuable tools to efficiently design a future rainbow trout specific SNP chip.

Results reported here represent the first study of EST-derived SNP validation in rainbow trout and demonstrate the utility of EST databases as an alternative approach for *de novo* SNP identification in species whose genome sequences are not yet available.

## Competing interests

The authors declare that they have no competing interests.

## Authors’ contributions

MB conceived and supervised the project, and drafted the manuscript. RG carried out the linkage maps and genetic diversity analysis, and revised the manuscript. CC performed EST-derived SNP identification and Sigenae SNP database management. DE carried out genotyping work. EQ contributed in designing the study, helped with interpretation of data analyses and revised the manuscript. All authors read and approved the final manuscript.

## Supplementary Material

Additional file 1 Minor sequence frequencies, heterozygosity and PIC values were indicated for all SNPs in the INRA Sigenae database (worksheet 1) and for the validation panel (worksheet 2). Allele frequencies were calculated by direct count of sequence depth for each allele, expected heterozygosities per locus (He) were estimated by He = 2pq and PIC values were estimated by PIC = H-2p^2^q^2^, where p and q are the sequence frequencies of the two alternate alleles. Click here for file

Additional file 2 Illumina quality design scores for the validation panel. Click here for file

Additional file 3 SNP allele frequencies. Allele frequencies for both alleles and minor allele frequencies (MAF) were shown for DH clonal lines (worksheet 1), INRA-SY (worksheet 2), INRA-SP (worksheet 3), NCCCWA (worksheet 4), all 3 populations (worksheet 5) and for true SNPs (worksheet 6). Allele frequencies were calculated by direct count in the raw genotype data file, expected heterozygosities per locus (He) were estimated by He = 2pq and PIC values were estimated by PIC = H-2p^2^q^2^, where p and q are the allele frequencies of the two alternate alleles. Click here for file

Additional file 4 Best k analysis. Results were shown for the five independent runs using a bur-in of 50,000 and 200,000 iterations. The best K value was determined using the non parametric test as previously described [29]. Click here for file

Additional file 5 SNP functional annotations. BlastX search results using EST contig (workseet 1) and SNP (worksheet 2) sequences as well as synonymous/non-synonymous SNP prediction results (workseet 3) were summerized. Click here for file

Additional file 6 INRA rainbow trout linkage maps. Newly integrated SNPs were highlighted in red. Click here for file

Additional file 7 List of duplicated markers and markers assigned in duplicated region. Click here for file
