# Supplementary figures and images for: Development and characterisation of an expressed sequence tags (EST)-derived single nucleotide polymorphisms (SNPs) resource in rainbow trout
Source: BMC Genomics. 2012 Jun 13;13:238. doi: 10.1186/1471-2164-13-238 (PMC3536561; doi:10.1186/1471-2164-13-238)

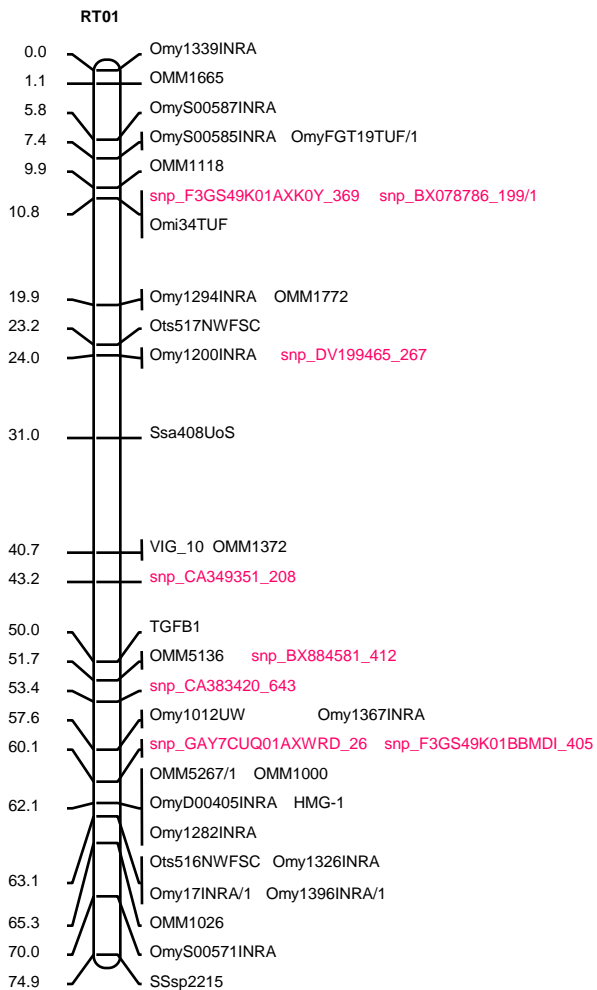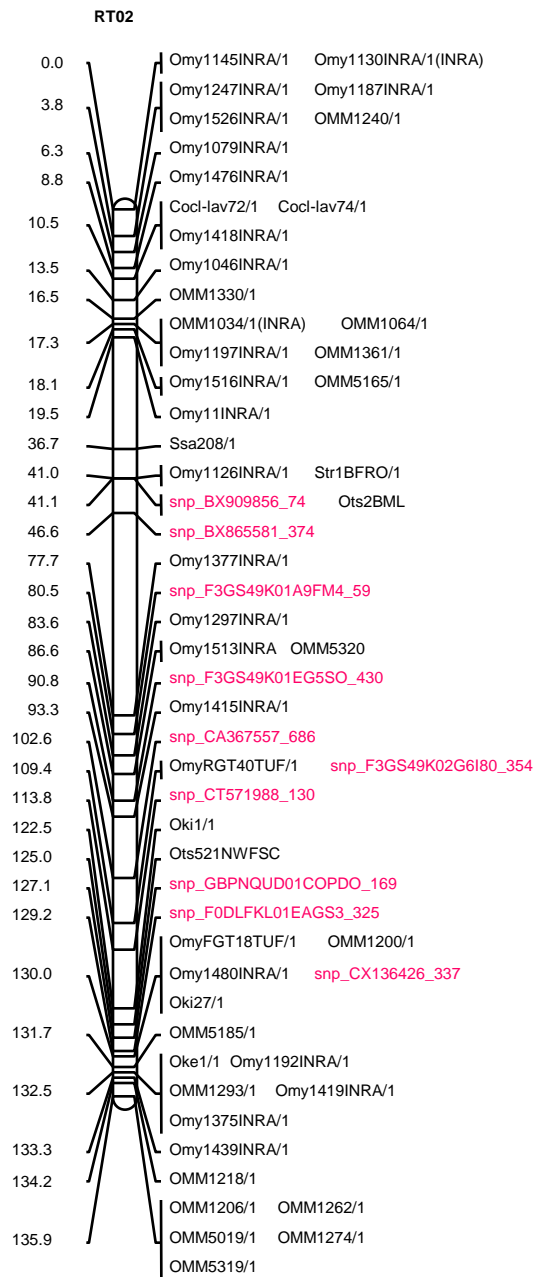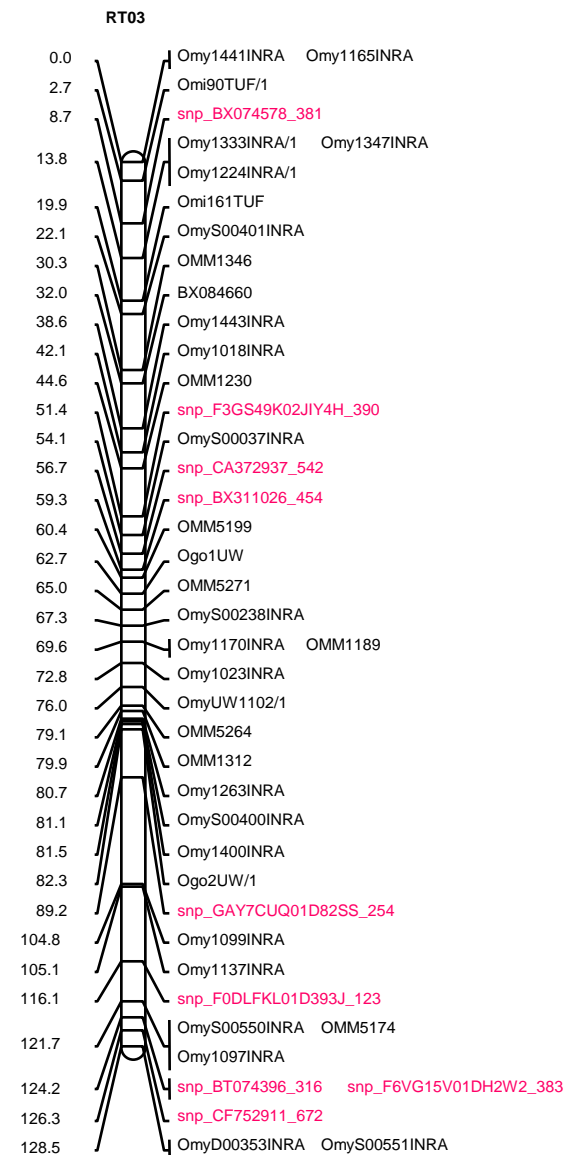

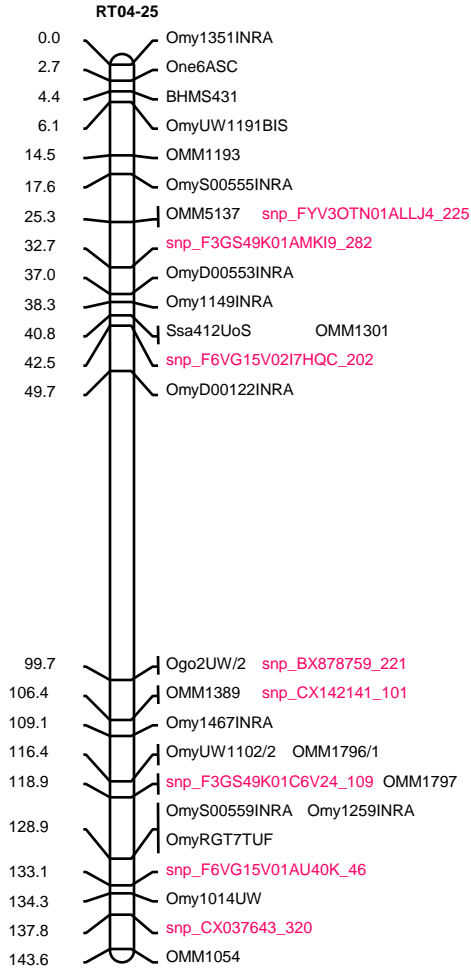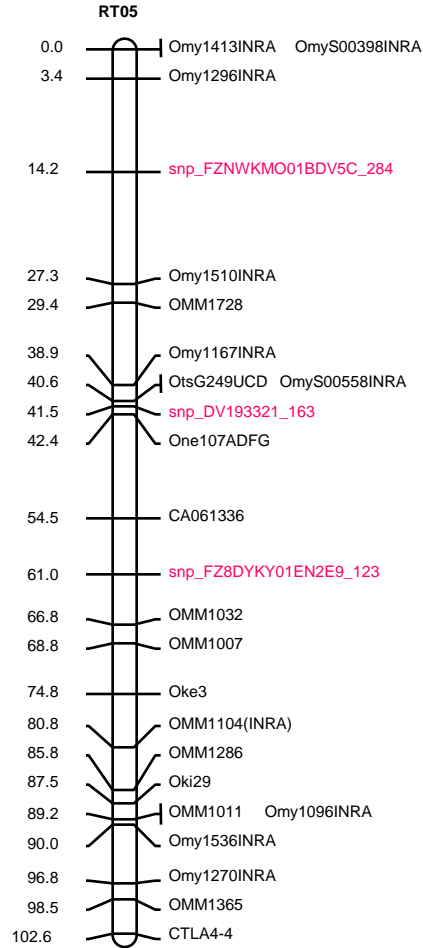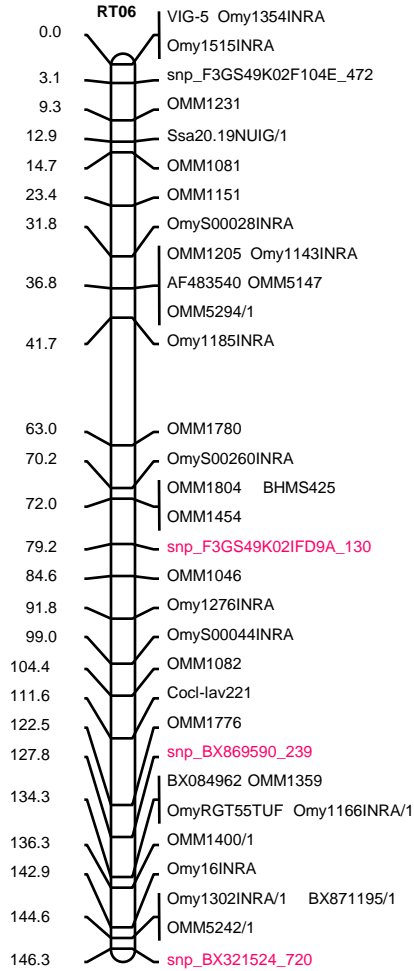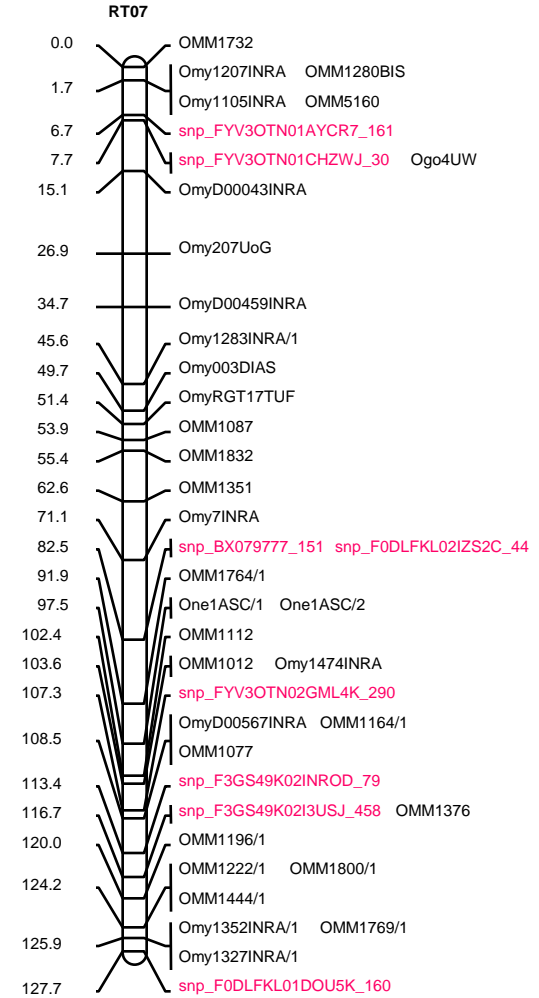

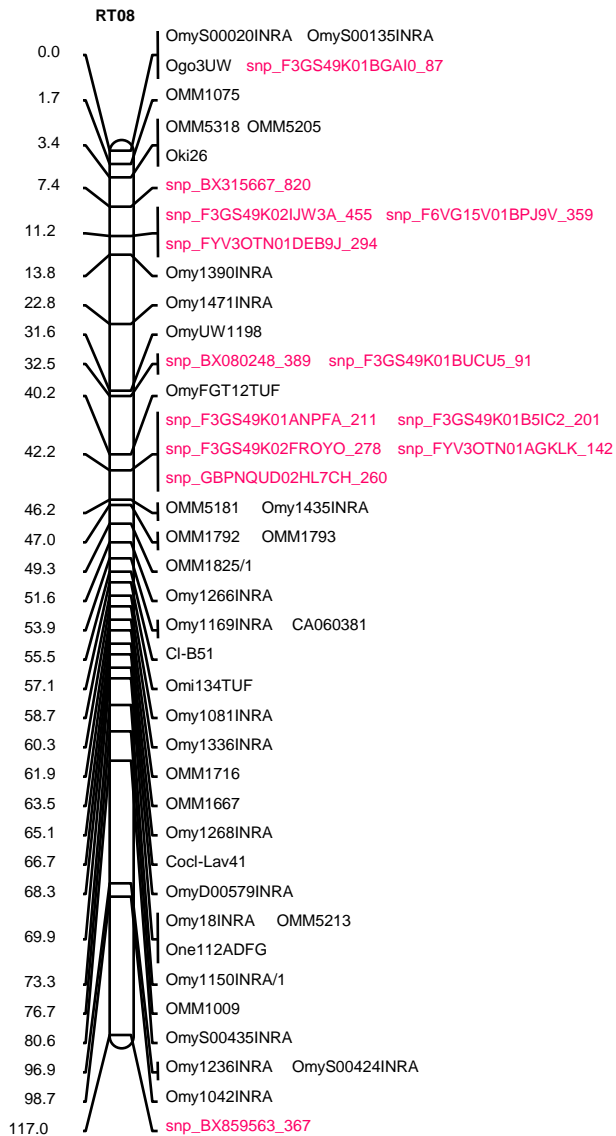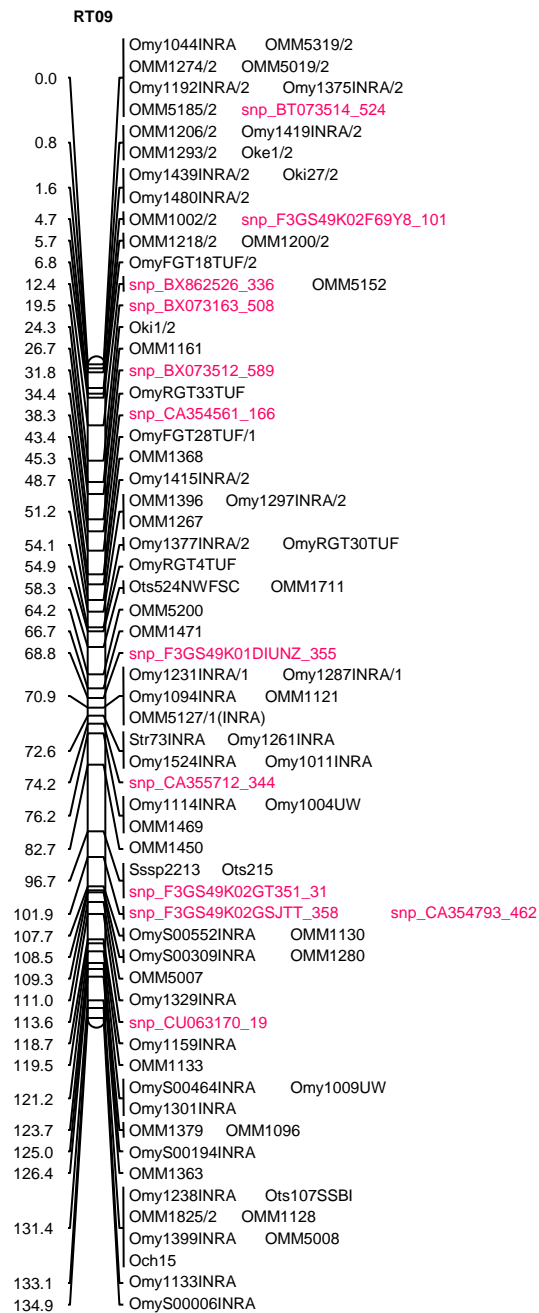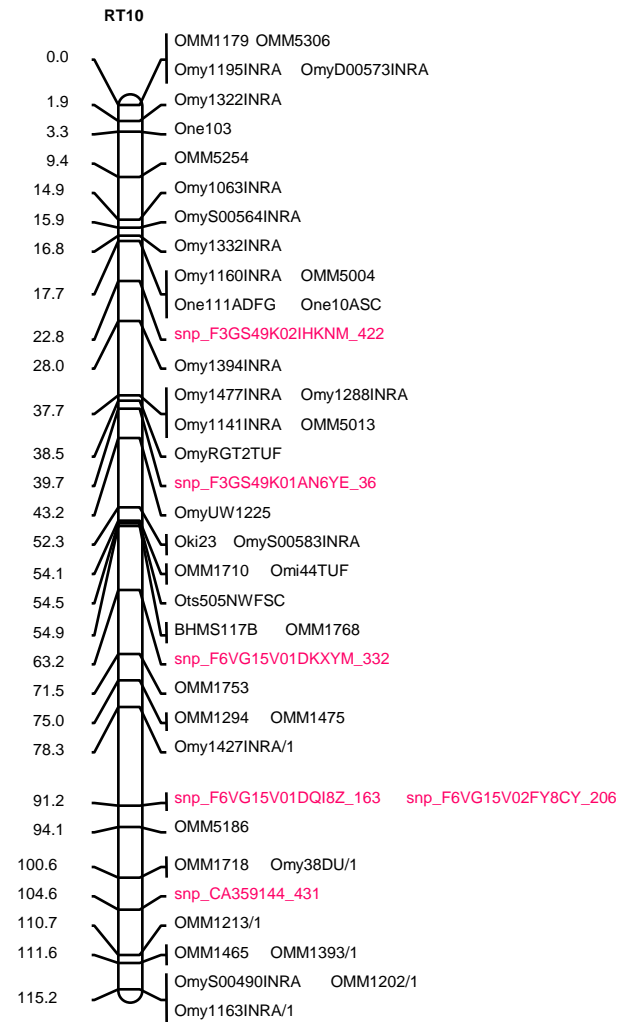

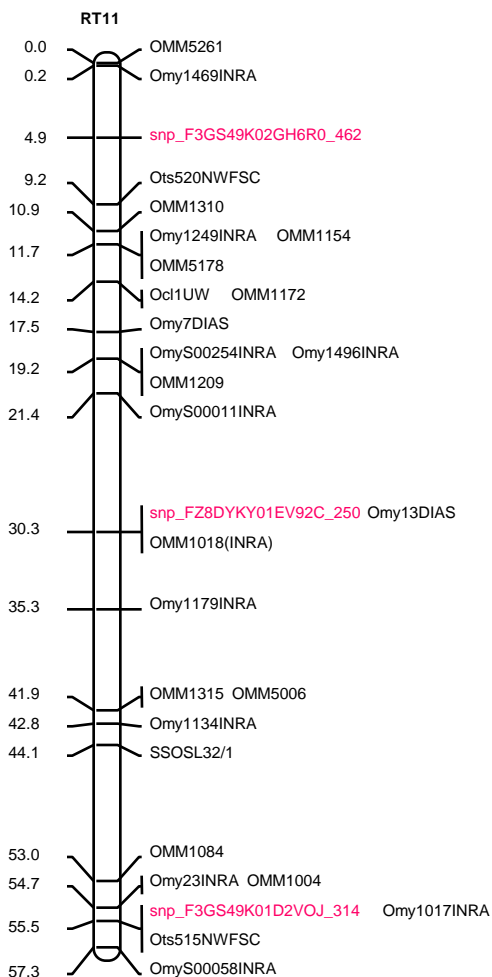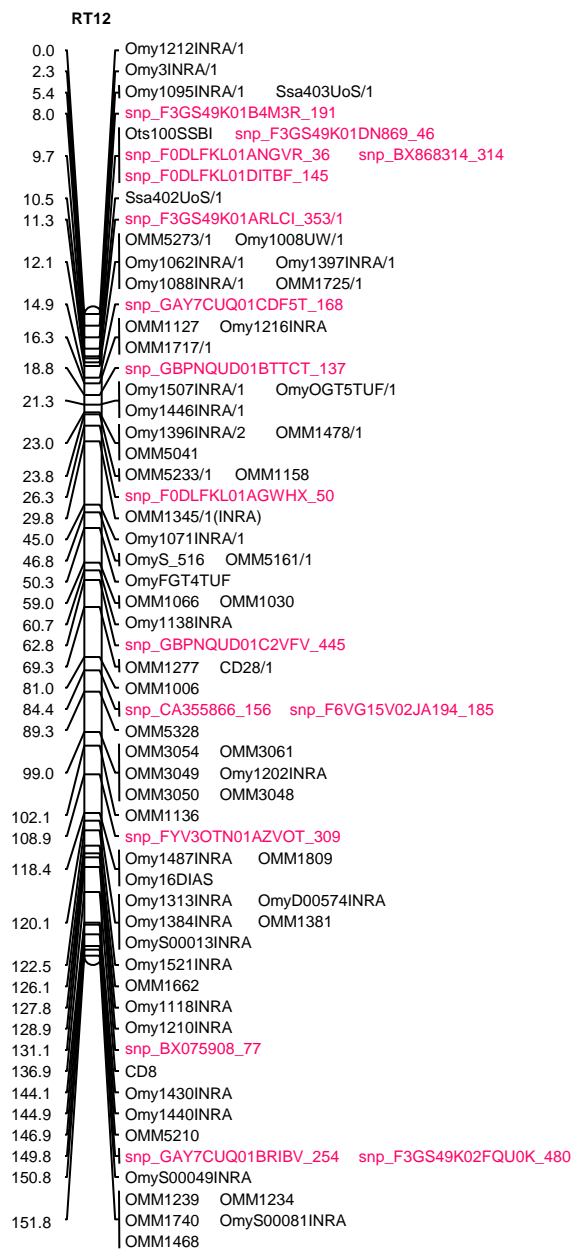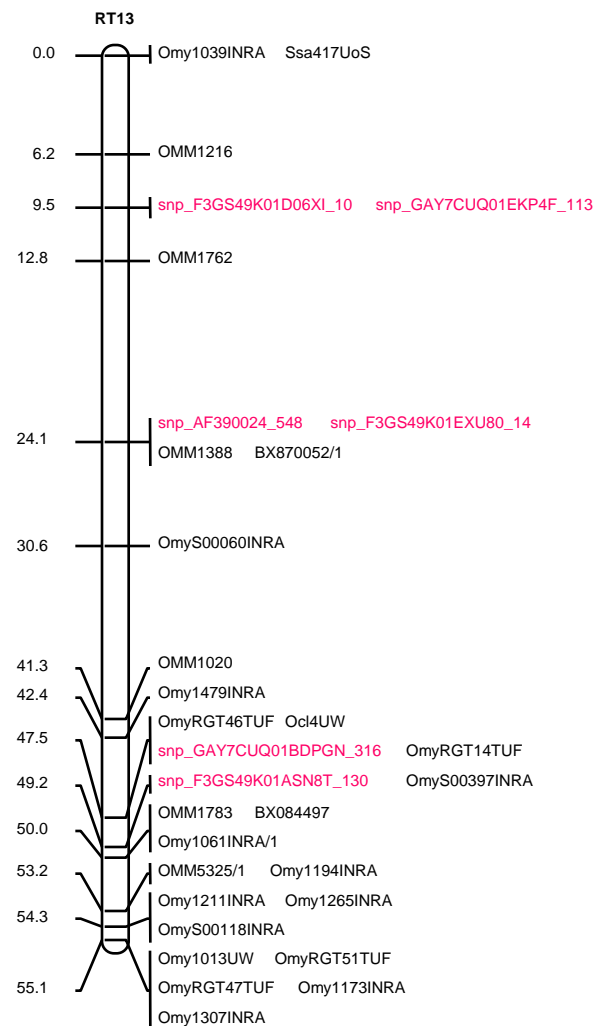

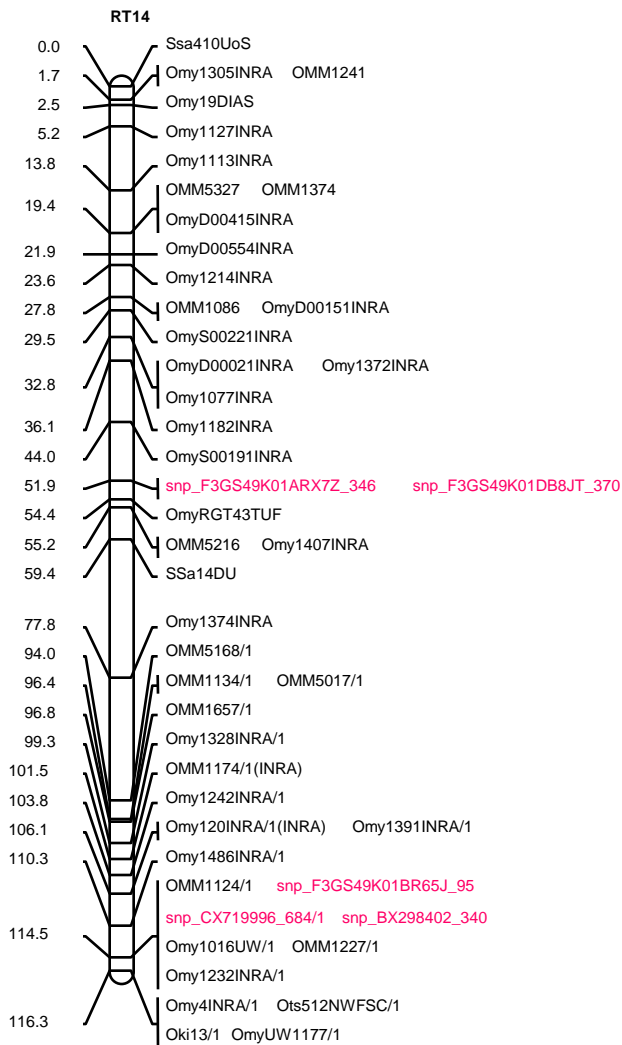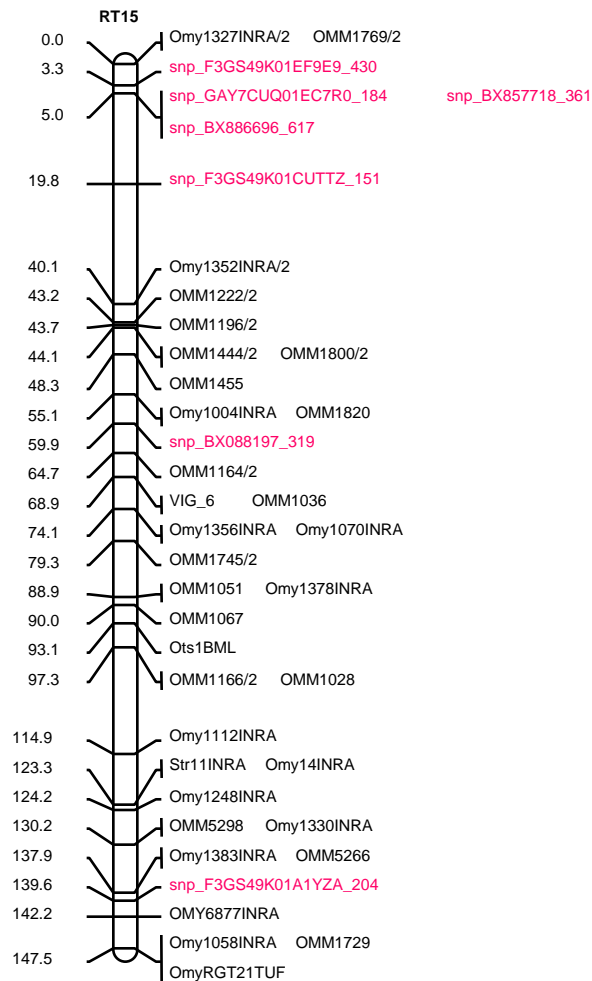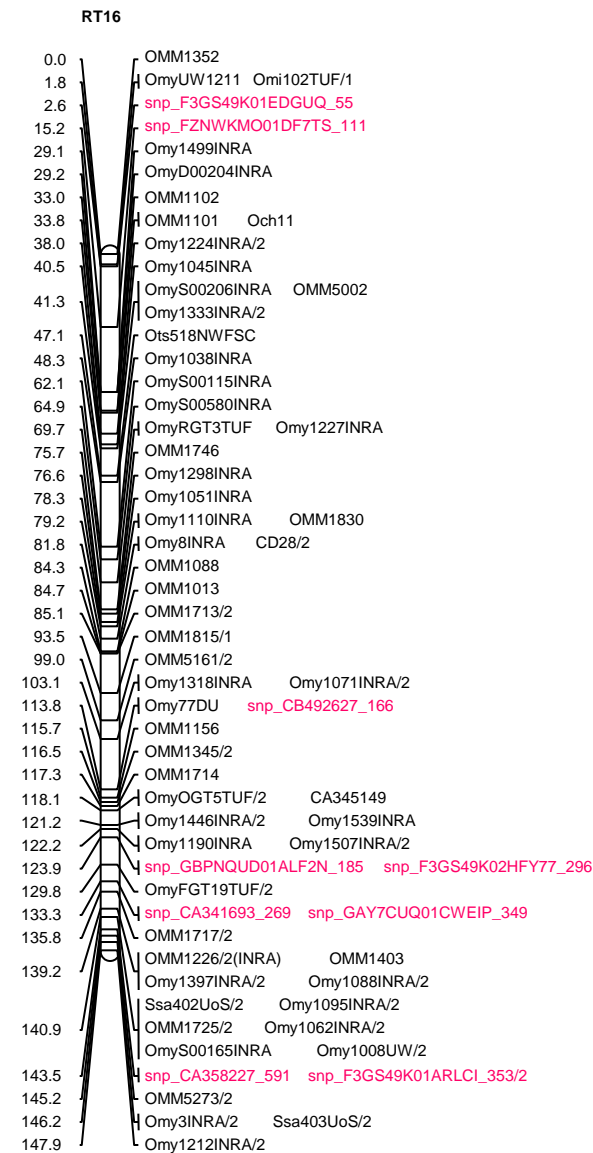

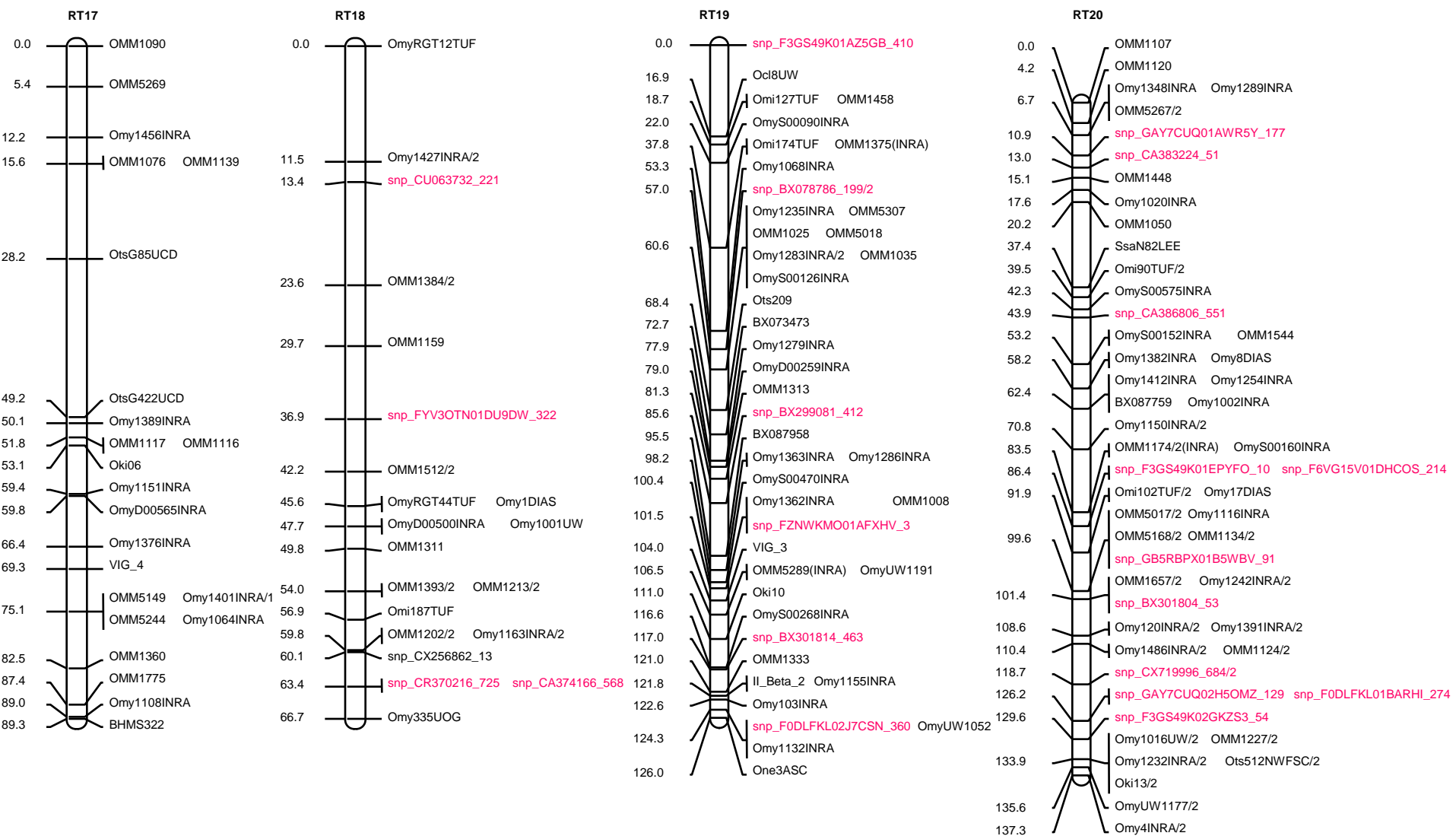

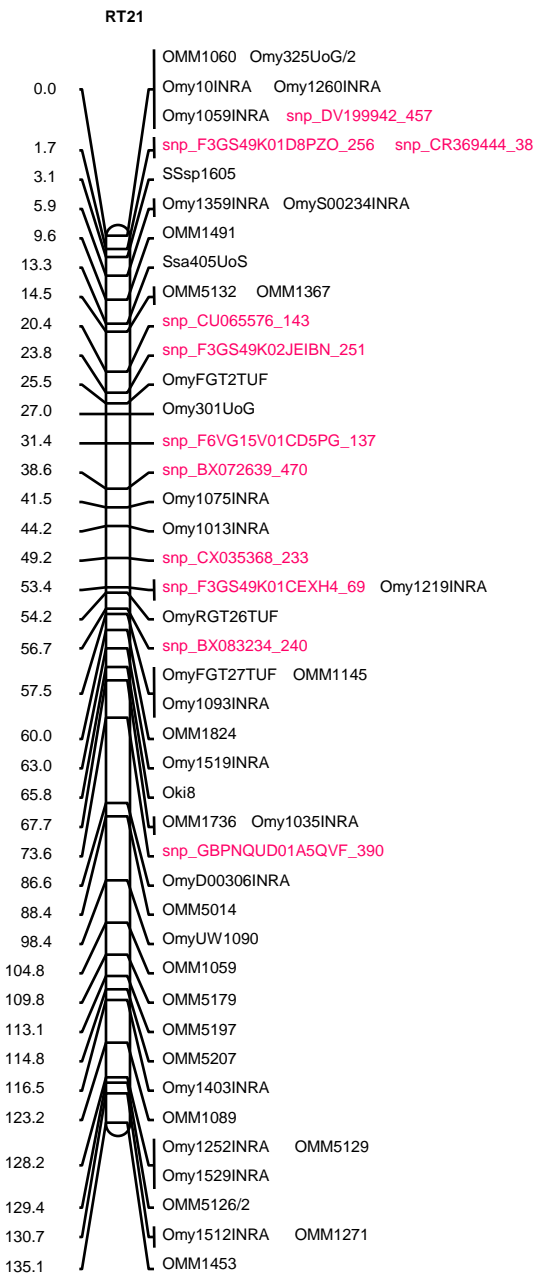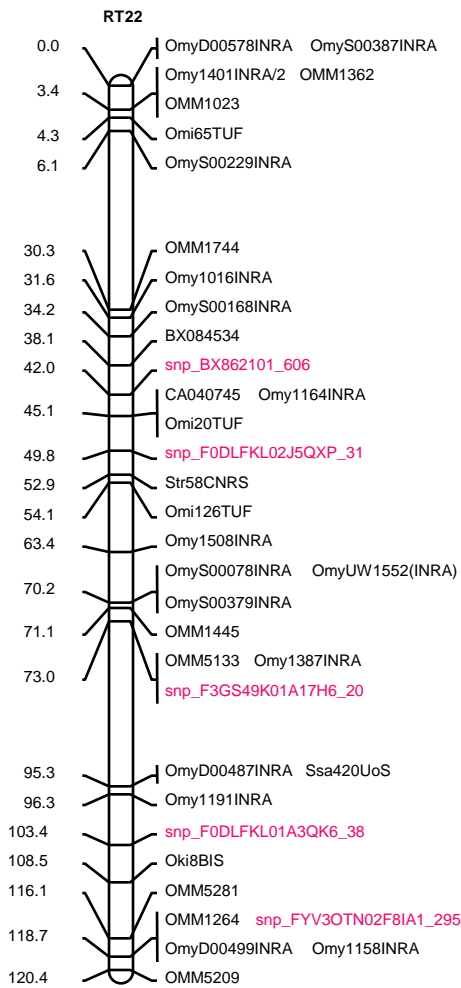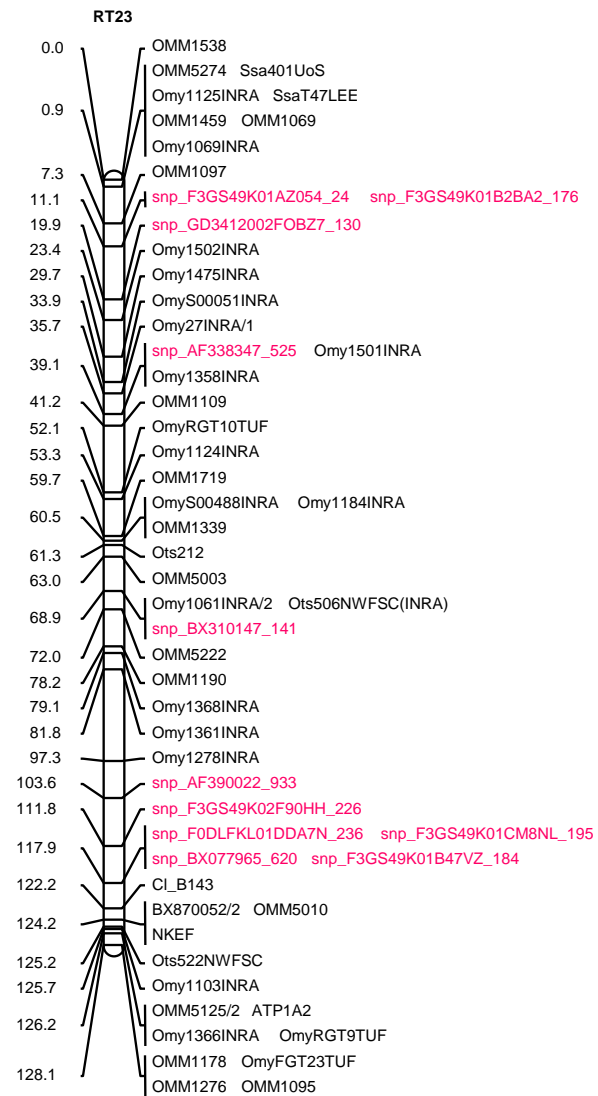

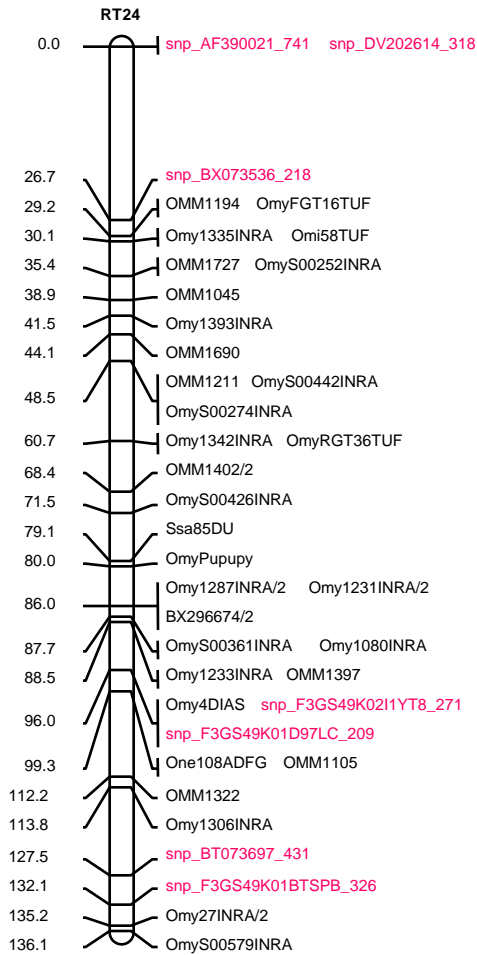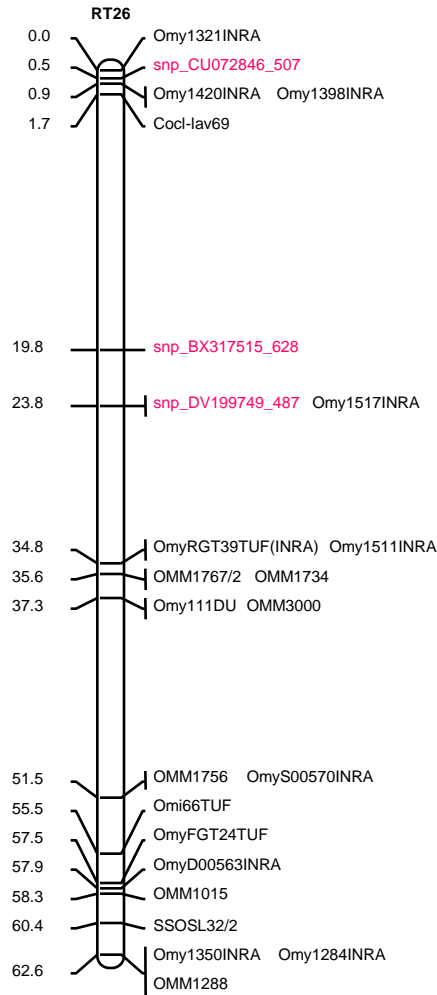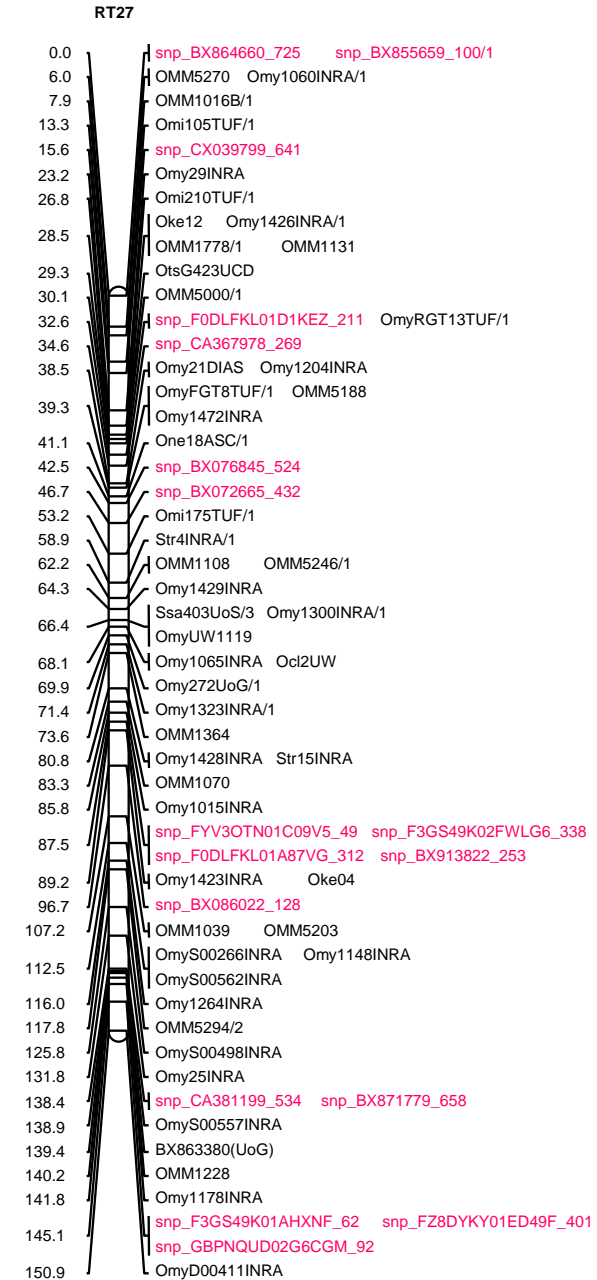

## RT29

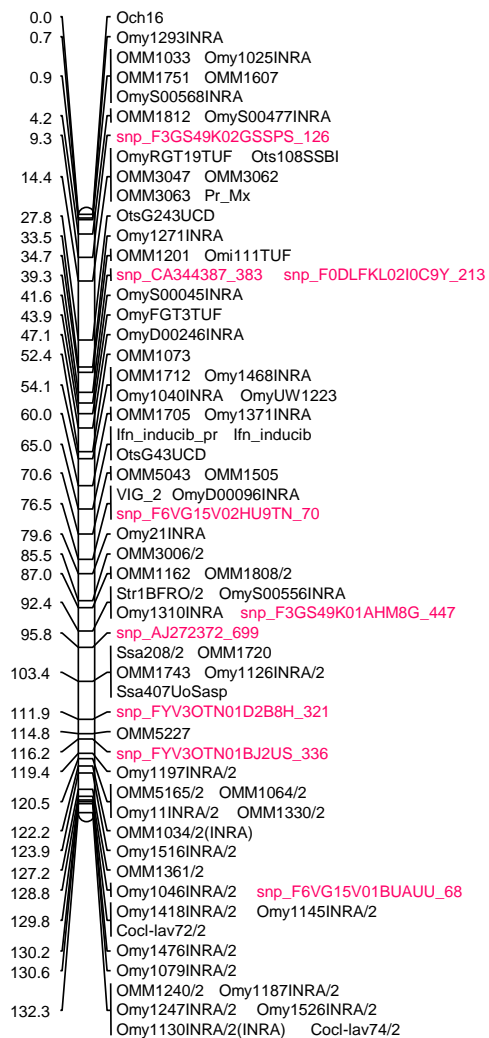

## RT30

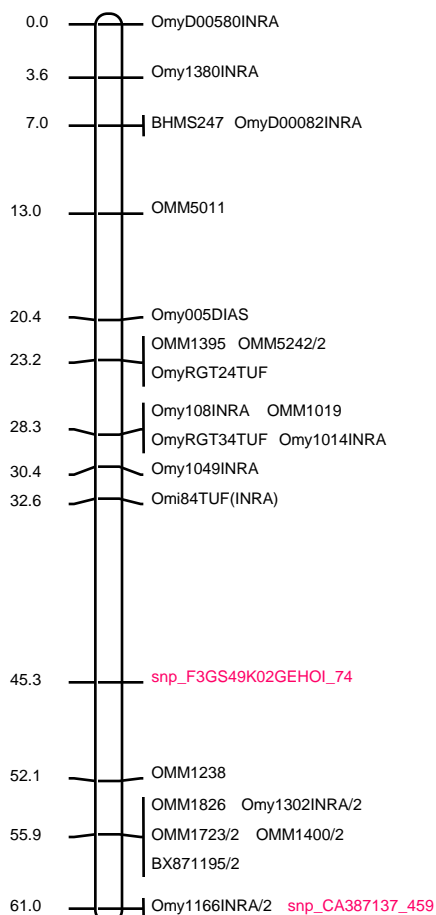

## RT31

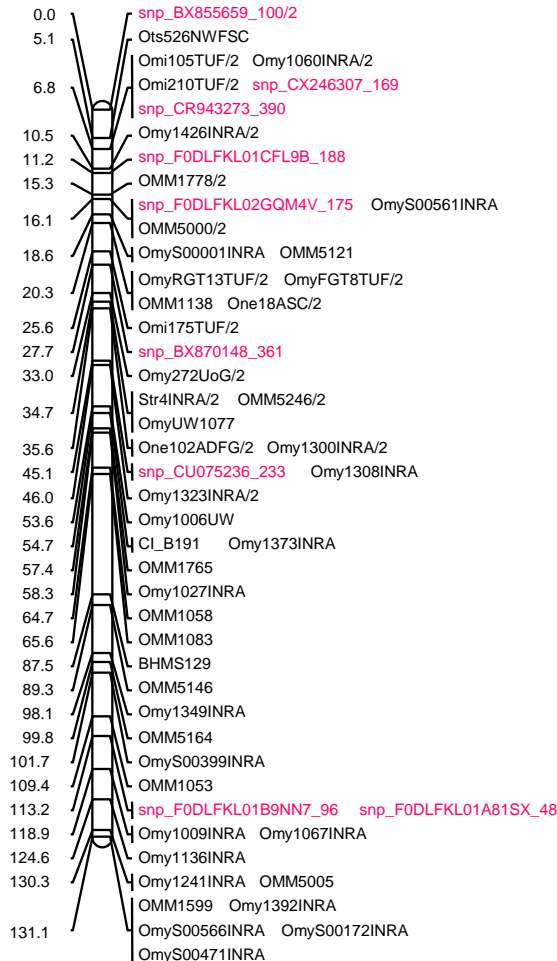

Supplement: Additional file 6 — INRA rainbow trout linkage maps. Newly integrated SNPs were highlighted in red. [file 1471-2164-13-238-S6.pdf]
